# Household polluting cooking fuels and adverse birth outcomes: An updated systematic review and meta-analysis

**DOI:** 10.3389/fpubh.2023.978556

**Published:** 2023-03-03

**Authors:** Mengrui Luo, Tiancong Liu, Changcheng Ma, Jianwei Fang, Zhiying Zhao, Yu Wen, Yang Xia, Yuhong Zhao, Chao Ji

**Affiliations:** ^1^Department of Clinical Epidemiology, Shengjing Hospital of China Medical University, Shenyang, China; ^2^Department of Otorhinolaryngology - Head and Neck Surgery, Shengjing Hospital of China Medical University, Shenyang, China; ^3^Department of Clinical Laboratory, Shengjing Hospital of China Medical University, Shenyang, China; ^4^Clinical Research Center, Shengjing Hospital of China Medical University, Shenyang, China

**Keywords:** cooking fuels, low birth weight, meta-analysis, preterm birth, small for gestational age, stillbirth

## Abstract

**Background and aim:**

The current study aimed to clarify the association between household polluting cooking fuels and adverse birth outcomes using previously published articles.

**Methods:**

In this systematic review and meta-analysis, a systematic literature search in PubMed, Embase, Web of Science, and Scopus databases were undertaken for relevant studies that had been published from inception to 16 January 2023. We calculated the overall odds ratio (OR) and 95% confidence interval (CI) for adverse birth outcomes [low birth weight (LBW), small for gestational age (SGA), stillbirth, and preterm birth (PTB)] associated with polluting cooking fuels (biomass, coal, and kerosene). Subgroup analysis and meta-regression were also conducted.

**Results:**

We included 16 cross-sectional, five case–control, and 11 cohort studies in the review. Polluting cooking fuels were found to be associated with LBW (OR: 1.37, 95% CI: 1.24, 1.52), SGA (OR: 1.48, 95% CI: 1.13, 1.94), stillbirth (OR: 1.38, 95% CI: 1.23, 1.55), and PTB (OR: 1.27, 95% CI: 1.19, 1.36). The results of most of the subgroup analyses were consistent with the main results. In the meta-regression of LBW, study design (cohort study: *P* < 0.01; cross-sectional study: *P* < 0.01) and sample size (≥ 1000: *P* < 0.01) were the covariates associated with heterogeneity. Cooking fuel types (mixed fuel: *P* < 0.05) were the potentially heterogeneous source in the SGA analysis.

**Conclusion:**

The use of household polluting cooking fuels could be associated with LBW, SGA, stillbirth, and PTB. The limited literature, observational study design, exposure and outcome assessment, and residual confounding suggest that further strong epidemiological evidence with improved and standardized data was required to assess health risks from particular fuels and technologies utilized.

## Introduction

Household air pollution (HAP) is often considered to be a major public health problem in low- and middle-income countries (LMICs) (1). HAP has a direct impact on human health and is an important risk factor for increased morbidity and mortality (2). The inefficient combustion of solid fuels (wood, coal, charcoal, dung, and crop waste) and kerosene in simple stoves and devices is a major source of HAP (3–5). Approximately 2.4 billion people cook mainly with polluting fuels (solid fuels and kerosene) globally (6). The inefficient combustion of polluting fuels often emits a high level of air pollutants (7). It is estimated that nearly 3 million people die from HAP exposure every year, the vast majority of whom live in LMICs (1). Research has shown that exposure to HAP is associated with chronic obstructive pulmonary disease, lung cancer, acute respiratory infections, cerebrovascular disease, ischemic heart disease, and adverse birth outcomes (8–11). The mechanism involved in the cardiorespiratory effect includes inflammation and oxidative stress induced by reactive oxygen and nitrogen species generated by inhaled pollutants (12, 13). HAP has also been associated with epigenetic adverse effects, which change DNA expression and potentiate the inflammatory effects of pollutants (14).

Perinatal morbidity and mortality are majorly associated with adverse birth outcomes, such as low birth weight (LBW), small for gestational age (SGA), stillbirth, and preterm birth (PTB) (15). For example, 21.9% of neonatal deaths were attributable to being born SGA (16). The leading causes of death in children under 5 years old were PTB complications (17). The risk factors of adverse birth outcomes include both individual (e.g., smoking, diet, antenatal depression, and antenatal care) and environmental factors (e.g., air pollution, occupational exposure, and pesticides) (18–22). For example, evidence suggests that the combustion of polluting cooking fuels emits high levels of air pollutants, such as fine particulate matter (PM_2.5_), carbon monoxide (CO), and polycyclic aromatic hydrocarbons (PAHs), which can influence fetal development (23, 24).

A growing number of epidemiological studies focus on the association between HAP and adverse birth outcomes. Two meta-analyses have pooled the effects of solid fuel use and adverse birth outcomes (25, 26). Both mainly focused on LBW and stillbirth, and only one study conducted a subgroup analysis for LBW (26). In addition, the study did not address the question of whether the strength of any association between polluting fuel use and the risk of adverse birth outcomes was affected by different types of polluting fuel (26). Since then, many new studies have been conducted, focusing on a broader range of fuel types and adverse birth outcomes, and the conclusion is still inconsistent. One cohort study (27), two case–control studies (28, 29), and six cross-sectional studies (30–35) suggested that polluting fuel use was related to increased risk of adverse birth outcomes, while other studies (23, 24, 36–47) failed to reveal a significant correlation between them. A recent review of the subject qualitatively assessed the impact of unclean cooking fuels on adverse birth outcomes, but the review was short of quantitative combined research data (48). The HAP field is evolving rapidly. Confirming or disputing earlier findings is vital in this circumstance. Furthermore, a timely review of research methods and results can assist or guide further research as well as public health policy. Based on these considerations, we carried out a systematic review and meta-analysis to determine whether HAP was associated with LBW, stillbirth, PTB, and SGA. We also explored whether the strength of any association between polluting fuel use and the risk of adverse birth outcomes was affected by different types of polluting fuel. Subgroup and meta-regression analyses were conducted to explore the sources of heterogeneity.

## Methods

The current systematic review and meta-analysis scrutinized the published adverse birth outcomes data related to polluting cooking fuel use from previous research publications using previously published search. We used the standard method of the preferred reporting items for systematic review and meta-analysis (PRISMA) protocols 2009 for conducting this review study (49). The study protocol was registered with PROSPERO (CRD42021269660).

### Search strategy

We searched the PubMed, Embase, Web of Science, and Scopus databases for articles reporting HAP and adverse birth outcomes published up to 16 January 2023. In the present review, HAP is defined as chemical, biological, and physical contamination of house air and derived from the use of polluting fuel (wood, dung, crop residues, charcoal, coal, and kerosene) for cooking (31). An infant weighing < 2,500 g was defined as LBW (23), and SGA referred to a baby weighing less than the 10th percentile for a certain gestational age (35). Stillbirth was defined as delivering a baby without any sign of life after 20 weeks of gestation (27, 50), while PTB was defined as an infant born before 37 weeks (24). The search process has been focused on the following terms: (indoor air pollution or household air pollution or cooking fuel or unclean fuel or solid fuel or biomass or wood or coal or kerosene or cooking) and (pregnancy outcome or pregnancy complications or low birth weight or premature birth or stillbirth or small for gestational age). The detailed search strategies are presented in Supplementary Table 1. We also scanned the reference lists of retrieved articles and previous meta-analyses to identify additional studies.

### Selection criteria

All studies obtained from the aforementioned resources were independently evaluated by two reviewers for inclusion and exclusion. First, the title and abstract of each study were reviewed, and the full text of the relevant studies was retrieved and assessed for inclusion eligibility. Studies were considered for inclusion if they were (1) original studies, (2) conducted in the human population, and (3) quantified the association between HAP exposure during pregnancy and adverse birth outcomes (LBW, SGA, stillbirth, and PTB). Studies were excluded for the following reasons: (1) the outcome was due to other factors (e.g., maternal age, educational level, and the house renovation) but not polluting cooking fuels (e.g., wood, dung, crop residues, charcoal, coal, and kerosene); (2) studies reported other outcomes those were not of interest; (3) the subjects from the control group were not exposed to clean fuels (e.g., electricity, liquid petroleum gas, natural gas, and biogas) or were exposed to polluting fuels; (4) studies did not report relative risk (RR) or odds ratio (OR); (5) conference abstract, letter, or protocol; (6) not published in English. Based on the inclusion and exclusion criteria, we excluded several unqualified studies from previous meta-analyses. The reasons for exclusion are as follows: reported the impact of other factors (e.g., cooking smoke and chimney stove) on adverse birth outcomes (*n* = 3) (51–53); the outcomes were not of interest (*n* = 2) (54, 55); did not report RR or OR (*n* = 3) (56–58); published in non-English (*n* = 2) (59, 60).

### Data extraction and quality assessment

Data from all included studies were independently extracted by two reviewers and cross-checked to avoid errors. The following information was extracted from the publications: author, publication year, location of the study, study population, study design, study period, exposure, outcome, covariates adjustment, the frequency distribution of exposure and outcome, comparator, exposure, and outcome assessment method. We also extracted risk estimates of association relating polluting cooking fuel to adverse birth outcomes for pooled analysis. RR or OR with precision [95% confidence interval (CI)] was extracted from included studies. The fully adjusted effect estimates were used for analysis when both unadjusted and adjusted estimates were provided.

The methodological quality of cohort and case–control studies was assessed by the Newcastle–Ottawa scale (range, 0–9 stars) (61). Each study was awarded stars based on three dimensions [selection, comparability, and outcome (cohort studies) or exposure (case–control studies)] (61). A study awarded seven or more stars was considered high quality (62), whereas one awarded three or fewer stars was considered low quality (63). The 11-item checklist was used for methodological quality assessment of cross-sectional studies, which had been recommended by the Agency for Healthcare Research and Quality (range, 0–11 scores) (64). A study with an eight or higher score was deemed to be of high quality and that with a three or lower score was of low quality (64).

### Statistical analysis

We assumed that RR was approximately equivalent to OR for our rare adverse birth outcomes (65, 66). To examine the association between polluting cooking fuels and adverse birth outcomes, we calculated ORs and corresponding 95% CIs. The *I*^2^ statistic was defined to assess the heterogeneity across studies. We reported the heterogeneity as low, moderate, or high with *I*^2^ values of 25, 50, or 75%, respectively (63). Publication bias was quantitatively assessed using Egger's tests. Moreover, the trim and fill method was adopted when publication bias existed. Meta-regression analysis was considered when there were 10 or more studies (67). Subgroup and meta-regression analyses were conducted by location (Asia, America, or Africa), type of study design (cohort, case–control, or cross-sectional), sample size (< 1,000 or ≥1,000), cooking fuel type [biomass fuels (wood, charcoal, crop residues, and animal dung), fossil fuels (coal and kerosene), or mixed fuels (biomass plus fossil fuels)], and outcome assessment method [direct assessment (measure and health/hospital records) or indirect assessment (maternal recall)]. Finally, sensitivity analyses were conducted to examine the influence of each study on the overall pooled estimate by the omission of each estimate one at a time (67). All the statistical analyses were performed using Stata statistical software version 14.0 (Copyright 1985–2015 StataCorp LP), and *P*-values < 0.05 were considered significant.

## Results

### Study selection

**Figure 1:**
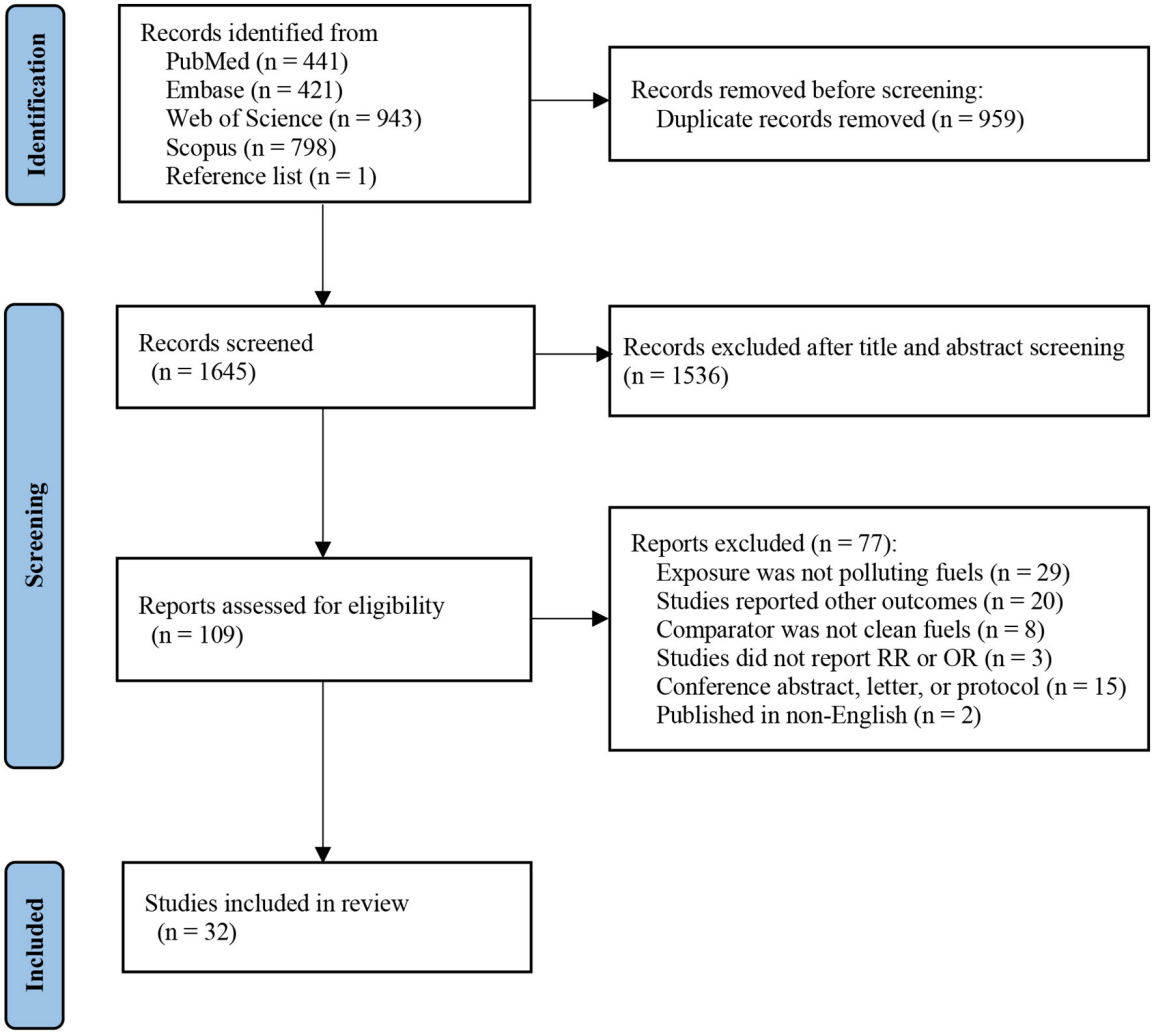
Flowchart of literature selection.

Figure 1 shows the literature screening processes in detail. In brief, 2,604 publications were identified through PubMed (*n* = 441), Embase (*n* = 421), Web of Science (*n* = 943), and Scopus (*n* = 798). An additional article was identified by a manual search of reference lists of included studies (*n* = 1). After excluding duplicates (*n* = 959), 1,536 records were excluded based on title and abstract. We obtained 109 articles for in-depth evaluation, of which 77 were excluded after reviewing the full text. Ultimately, 32 studies were enrolled in our final systematic review and meta-analysis.

### Study characteristics

A summary of the characteristics of 32 publications is presented in Table 1. A total of 23 studies were conducted in Asia (9–11, 23, 24, 30–41, 43, 47, 68–71), three were conducted in America (28, 42, 72), five in Africa (29, 44–46, 73), and one study used a sample from more than one continent (27). Study designs included cohort studies (*n* = 11) (10, 11, 24, 27, 37, 40, 42, 44, 45, 47, 68), case–control studies (*n* = 5) (28, 29, 43, 70, 72), and cross-sectional studies (*n* = 16) (9, 23, 30–36, 38, 39, 41, 46, 69, 71, 73). Supplementary Table 2 quantitatively describes the association between exposure and outcome. Exposure to HAP during pregnancy was assessed through an interview or questionnaire survey by inquiring about the main cooking fuel type. In total, 13 studies focused on HAP from biomass fuels on adverse birth outcomes (9–11, 28, 35, 36, 38, 42, 45, 68, 70, 72, and 73). Six studies investigated biomass and fossil fuels and their impact on outcomes (24, 29, 30, 37, 40, and 71). In total, 13 studies classified fuels as polluting (biomass and fossil) or clean (23, 27, 31–34, 39, 41, 43, 44, 46, 47, 69) fuels. The comparator group was either exposure to clean fuels (electricity, liquid petroleum gas, natural gas, and biogas) (*n* = 29) (9, 10, 23, 24, 27–41, 43–47, 68, 69, and 71–73) or “no exposure” to polluting fuels (*n* = 3) (11, 42, and 70). In addition, three studies also directly measured the concentrations of kitchen air pollutants, such as CO (28), PM_2.5_ (40), and inhalable particles (PM_10_) (38), and quantified an exposure–response relationship between air pollutants and adverse birth outcomes. Seven studies directly measured birth outcomes (11, 24, 29, 33, 34, 36, and 70), eight relied on maternal recall (9, 30, 31, 38, 39, 41, 47, and 71), and 12 collected data from medical/health records (10, 28, 32, 35, 37, 40, 42, 43, 45, 68, 72, and 73), while the other five used a combination of maternal recall and medical records (23, 27, 44, 46, and 69). A total of 21 studies only reported one adverse birth outcome (9, 10, 23, 27–34, 40–43, 46, 47, 69–71, and 73), and 11 reported two or more outcomes (11, 24, 35–39, 44, 45, 68, and 72).

**Table 1:** Study characteristics of the included studies.

**Author**	**Year**	**Location**	**Study population**	**Study design**	**Study period**	**Exposures**	**Outcomes**	**Covariates adjustment**
Mishra et al. (9)	2005	India	18,567 ever-married women included in India's 1998–99 National Family Health Survey (NFHS-2)	Cross-sectional study	1998–1999	Biomass (wood, animal dung, or crop residues)	Stillbirth	Tobacco smoke, woman's nutritional status, socioeconomic status, household conditions, urban/rural residence, and geographic region
Siddiqui et al. (68)	2005	Pakistan	1,404 pregnant women from a maternal child health surveillance program from communities in Nara, Kotdiji, and Bilal colonies	Prospective cohort study	2000–2001	Wood	LBW Stillbirth	LBW: Mother's BMI, Gravida status, prenatal vaccine, SES score, location (rural/location); Stillbirth: location (rural/urban)
Siddiqui et al. (10)	2008	Pakistan	634 women who had a singleton live birth in Rehri Goth	Retrospective cohort study	2000–2002	Wood	LBW	Prenatal examination in hospital, assessment day of newborn, maternal BMI, and parity and gravidity
Tielsch et al. (11)	2009	India	11,728 live-born singleton infants in two rural blocks in southern Tamil Nadu	Prospective cohort study	from birth through 6 months	Wood or dung	LBW SGA PTB Stillbirth	Number of children < 5 years of age in the household, place of delivery, roof material, religion, maternal night blindness, maternal age, maternal education, parity, television/radio ownership, electricity in the household, and SHTS exposure
Sreeramareddy et al. (69)	2011	India	47,139 singleton births in 2005–06 India Demographic Health Survey (DHS)	Cross-sectional study	2005–2006	High pollution fuels (wood, straw, animal dung, crop residues, kerosene, coal, and charcoal)	LBW	Sex of the baby, birth order, age at childbirth, maternal smoking, educational status, BMI, hemoglobin, religion, wealth index, type of residence (urban/rural)
Yucra et al. (72)	2011	Peru	190 singleton births in public hospitals from Abancay and Huancavelica	Case-control study	between January 2008 and May 2009	Biofuel	LBW PTB	Maternal age, education level, BMI, parity
Abusalah et al. (70)	2012	Gaza Strip	446 live singleton infants of Mbarak Hospital and Maternal Hospital of Shifa Medical Center	Case-control study	May–June and July–August 2007	Wood	LBW	Parents' education, occupation and residence, income, consanguinity, and BMI
Amegah et al. (73)	2012	Ghana	592 singleton births at KBTH Maternity Department	Cross-sectional study	–	Charcoal	LBW	Age, social class, marital status and gravidity of mothers, and sex of neonate
Epstein et al. (71)	2013	India	14,850 singleton births in India's National Family Health Survey (NFHS-3)	Cross-sectional study	2005–2006	Biomass; Kerosene; Coal	LBW	Maternal literacy, highest level of education obtained, highest year of education, literacy
Wylie et al. (36)	2014	Central and East India	1,744 pregnant women recruited at the time of delivery in Jharkhand and Chhattisgarh state	Cross-sectional study	From December 2006 to December 2007 in Jharkhand and from June 2007 to May 2008 in Chhattisgarh	Wood	LBW SGA PTB Stillbirth	LBW: propensity score, cohort (Jharkhand vs. Chhattisgarh), maternal age, BMI gravidity, hemoglobin at delivery, and time spent cooking; SGA: propensity score, cohort (J vs. C), gravidity, hemoglobin at delivery, fever in week prior to delivery and time spent cooking; PTB: propensity score, cohort (J vs. C), maternal age, BMI, gravidity, hypertension at delivery, hemoglobin at delivery, presence of windows, and time spent cooking; Stillbirth: propensity score
Yucra et al. (28)	2014	Peru	202 full-term births in public hospitals and health centers from Huancavelica and Junin	Case-control study	From August 2011 to May 2012	Biofuel	SGA	Education level and parity
Demelash et al. (29)	2015	South-East Ethiopia	387 full-term singleton births in the four governmental hospitals in Bale zone	Case-control study	From April 1 to August 30, 2013	Firewood; Kerosene; Animal dung	LBW	Unreported
Jiang et al. (37)	2015	Lanzhou, China	9,895 singleton live births from a birth cohort study conducted during 2010–2012 at the Gansu Provincial Maternity & Child Care Hospital (GPMCCH)	Cohort study	2010–2012	Coal; Biomass	LBW SGA	SGA: maternal age, education, family income, maternal weight gain, vitamin supplement during pregnancy, preeclampsia, cesarean section, parity, smoking, and ventilation; LBW: additional adjustment for gestational week
Mukherjee et al. (38)	2015	India	404 premenopausal women aged between 21 years and 43 years from eight villages of different districts in eastern India	Cross-sectional study	–	Biomass	LBW Stillbirth	SES, ETS, BMI, among other factors
Patel et al. (27)	2015	India, Pakistan, Kenya, Zambia, Guatemala	65,912 singleton pregnancies in rural communities in five low and lower middle-income countries	Prospective cohort study	May 2011 and Oct 2012	Polluting fuel (kerosene, charcoal, coal, wood, straw, crop waste, dung)	Stillbirth	Global network site
Haider et al. (30)	2016	Bangladesh	8,753 live births in the 2011 Bangladesh Demographic and Health Survey (BDHS)	Cross-sectional study	–	Coal; Wood; Straw/Crop	LBW	Unreported
Khan et al. (39)	2017	Bangladesh	22,789 singleton live-born children from Bangladesh Demographic and Health Survey	Cross-sectional study	2007–2014	Solid fuel (coal, lignite, charcoal, wood, straw/shrubs/grass, agricultural crop, animal dung, and others)	LBW Stillbirth	Maternal age, education, place of residence, region, socioeconomic status, breastfeeding and child sex
Balakrishnan et al. (40)	2018	India	1,121 singleton births from primary health care centers and urban health posts in Tamil Nadu	Prospective cohort study	2010–2015	Kerosene; Biomass	LBW	None
Nisha et al. (41)	2018	Bangladesh	27,237 singleton pregnancies from the Bangladesh Demographic and Health Surveys (BDHS) 2004, 2007, 2011, and 2014	Cross-sectional study	2004–2014	Polluting fuel (kerosene, coal/lignite, charcoal, wood, straw/shrubs/grass, agricultural crop, and animal dung)	Stillbirth	Maternal age at birth, maternal education, birth order, maternal BMI, place of residence, wealth index, maternal working status, location of kitchen, and year of survey
Suryadhi et al. (31)	2019	Indonesia	36,726 singleton births from 2012 Indonesian Demographic Health Survey (IDHS)	Cross-sectional study	Between May 7 and July 31, 2012	Solid fuel (coal, lignite, charcoal, wood, or straw/shrubs/grass)	LBW	Child's age, child's sex, mother's age, mother's education, residential area, and environmental tobacco smoke
Fleisch et al. (42)	2020	United States	1,223 women from the New Hampshire Birth Cohort Study	Cohort study	December 2018 to December 2019	Wood	SGA	Maternal age, education, race/ethnicity, pre-pregnancy BMI, cohort enrollment season, neighborhood wood stove use, home distance to nearest major roadway, and child's sex
Gurung et al. (32)	2020	Nepal	50,209 deliveries in the selected 12 public hospitals	Cross-sectional study	From 1 July 2017 to 29 August 2018	Polluted fuel	PTB	Unreported
Basel and Singh (43)	2020	Nepal	369 singleton births in health facilities of Dang district	Case-control study	July 2018 to March 2019	Firewood; Kerosene	LBW	Unreported
Gautam Paudel et al. (33)	2020	Nepal	60,695 births in 12 public hospitals	Cross-sectional study	From 1 July 2017 to 29 August 2018	Polluted fuel	SGA	Maternal age, education, ethnicity, smoking, anyone in the same house smokes, type of fuel used for cooking, parity, deliveries, anemia, antepartum hemorrhage, antenatal care visit, time of first ANC visit, delivery preparation, and sex of baby
Weber et al. (44)	2020	Accra, Ghana	819 pregnant women from the outpatient clinics of Maamobi General Hospital and Ridge Regional Hospital	Cohort study	Between July 2012 to March 2014	Polluting fuel (firewood, charcoal, kerosene, or crop residue/sawdust)	LBW SGA PTB	BMI, maternal age, maternal education, and SES
Hussein et al. (45)	2020	Northern region of Ghana	1,323 pregnant women in four hospitals located in Northern Region of Ghana	Prospective cohort study	From July 2018 through May 2019	Firewood; Charcoal	LBW SGA PTB	LBW: maternal malaria, kitchen hours, number of people cooked for, use of disinfectants; SGA: maternal BMI at first visit, anemia, use of disinfectants; PTB: Maternal malaria, kitchen hours, number of people cooked for, number of cooking sessions per day
Chaudhary et al. (34)	2021	Nepal	4,000 live births at Universal College of Medical Sciences, a 700-bedded tertiary care hospital situated in province five of Western Nepal	Cross-sectional study	–	Solid fuel	SGA	Age group, sex of babies, maternal age, maternal sleep, education, high carb snack, solid fuel use, smoking, environmental tobacco smoking, pregnancy-induced hypertension, gestational diabetes, cardiovascular diseases, polyhydramnios, hypothyroid, and anemia
Islam et al. (23)	2021	India	93,721 full-term singleton births from the fourth round of the National Family Health Survey (NFHS-4)	Cross-sectional study	2015–2016	Unclean cooking fuels (wood, agricultural by-products/residues/wastes, straw/shrubs/grass, animal dung, kerosene, coal/lignite, charcoal, and other fuels)	LBW	Environmental tobacco smoke, sex of the child, birth order of the child, mother's age at childbirth, mother underweight, mother's anemia status, antenatal care during pregnancy, pregnancy intention, mother's tobacco use, mother's education, social groups, wealth quintiles, and area of residence
Kanno et al. (46)	2021	Ethiopia	10,014 singleton births from the 2016 Ethiopian Demographic Health Survey (EDHS)	Cross-sectional study	–	high- pollution cooking fuels (wood, straw, animal dung, crop residues, kerosene, coal, and charcoal)	LBW	Child factors (i.e., gender of the baby and birth order), maternal factors (i.e., anemia level, BMI, age at first childbirth, chat chewing, alcohol drinking, education, and pregnancy intention) and sociodemographic factors (i.e., place of residence [urban/ rural], wealth index, sex of head of the household)
Vakalopoulos et al. (35)	2021	Sri Lanka	445 live births at maternity clinics in rural communities in Sri Lanka's Central Province	Cross-sectional study	Between August and September 2019	Biomass fuel	LBW SGA	Income, education, area, incense, vaporizer, second-hand tobacco smoke, and chimney
Lu et al. (47)	2022	China	30,735 preschoolers from more than 200 kindergartens at different administrative areas in six cities of China (Urumqi, Taiyuan, Nanjing, Shanghai, Chongqing and Changsha)	Retrospective cohort study	Between December 2010 and January 2012	Coal/wood	LBW	Child's sex, birth season, parental atopy, maternal occupation during pregnancy, parental smoking during pregnancy, window condensation, size of the home (m^2^), keeping cats, keeping dogs, city-level data, indoor mold/dampness, exposure to outdoor temperature and PM_10_, SO_2_, and NO_2_
Pan et al. (24)	2022	Guangxi, China	10,329 live births in Guangxi Zhuang Birth Cohort (GZBC) in Guangxi Zhuang Autonomous Region	Prospective cohort study	Between June 2015 and April 2018	Coal; Wood	LBW SGA PTB	Maternal age, birthplace, study county, occupation, pre-pregnancy BMI, alcohol drinking, regular physical activity, daily use of folic acid/multivitamin, chronic diseases, thalassemia, gravidity, parity, frequency of antenatal care visit, infant sex, and birth season, passive smoking, raising animals, dyeing hair, whether ventilation equipment in the kitchenware installed, whether live near factory/main roads, and occupational exposure

LBW, low birth weight; SHTS, second-hand tobacco smoke; PTB, preterm birth; SGA, small for gestational age; BMI, body mass index; SES, socioeconomic status; ANC, antenatal care; ETS, environmental tobacco smoke.

Detailed quality assessments are shown in Supplementary Tables 3–5. In general, six cohort studies were of high quality, while the rest were of moderate quality.

### Pooled analysis

**Figure 2:**
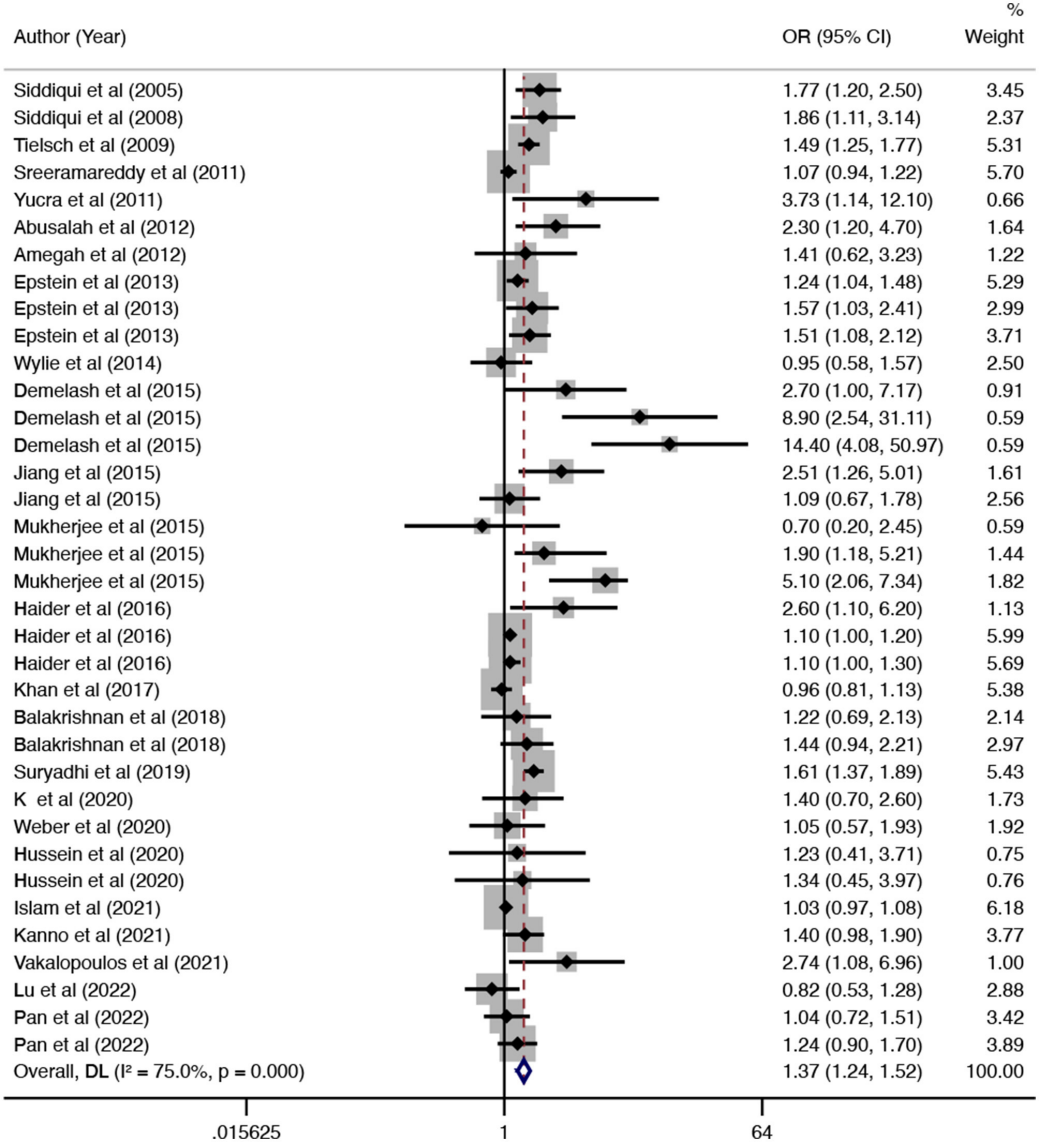
Forest plot showing the effect of polluting cooking fuels on low birth weight (LBW).

**Figure 3:**
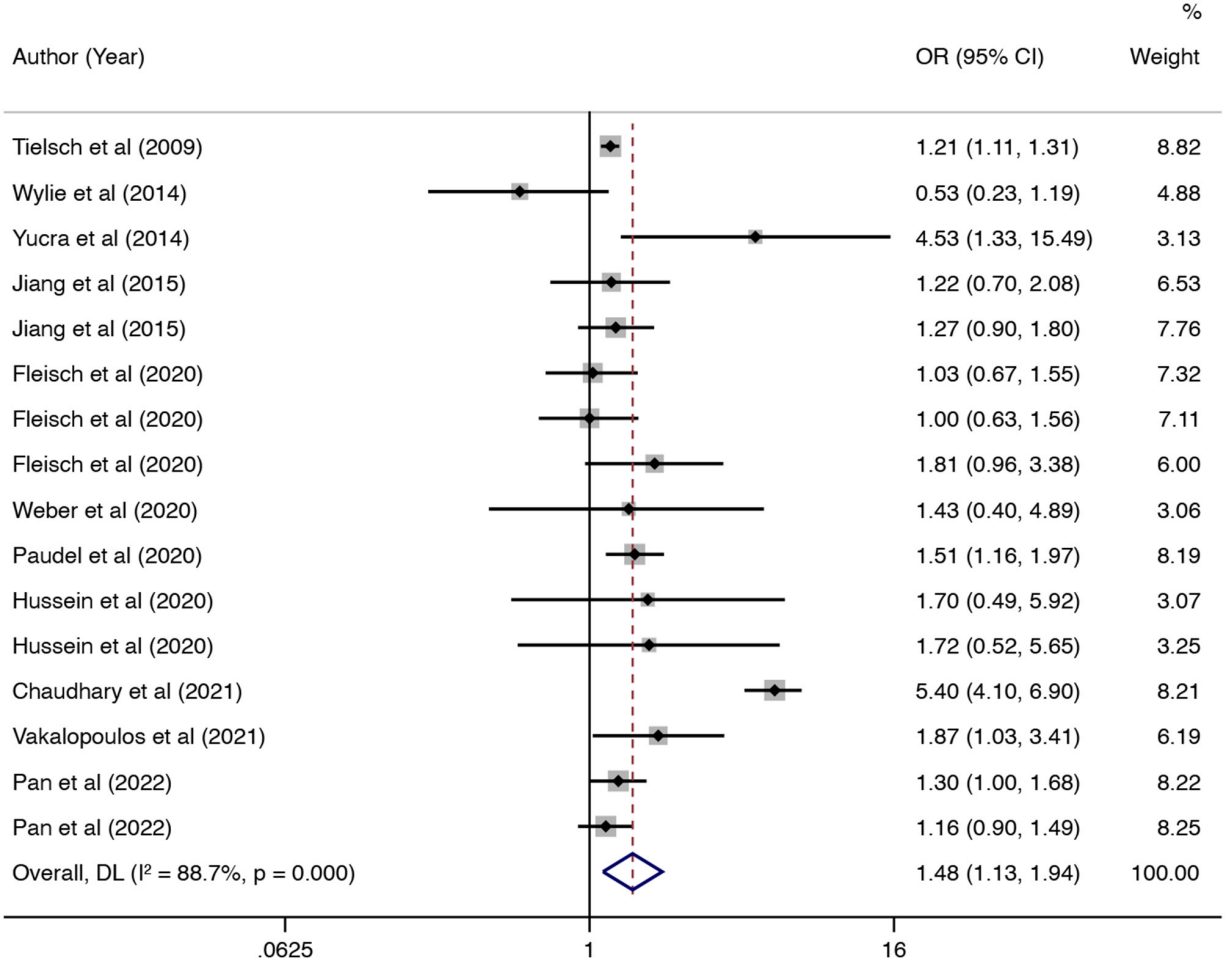
Forest plot showing the effect of polluting cooking fuels on small for gestational age (SGA).

**Figure 4:**
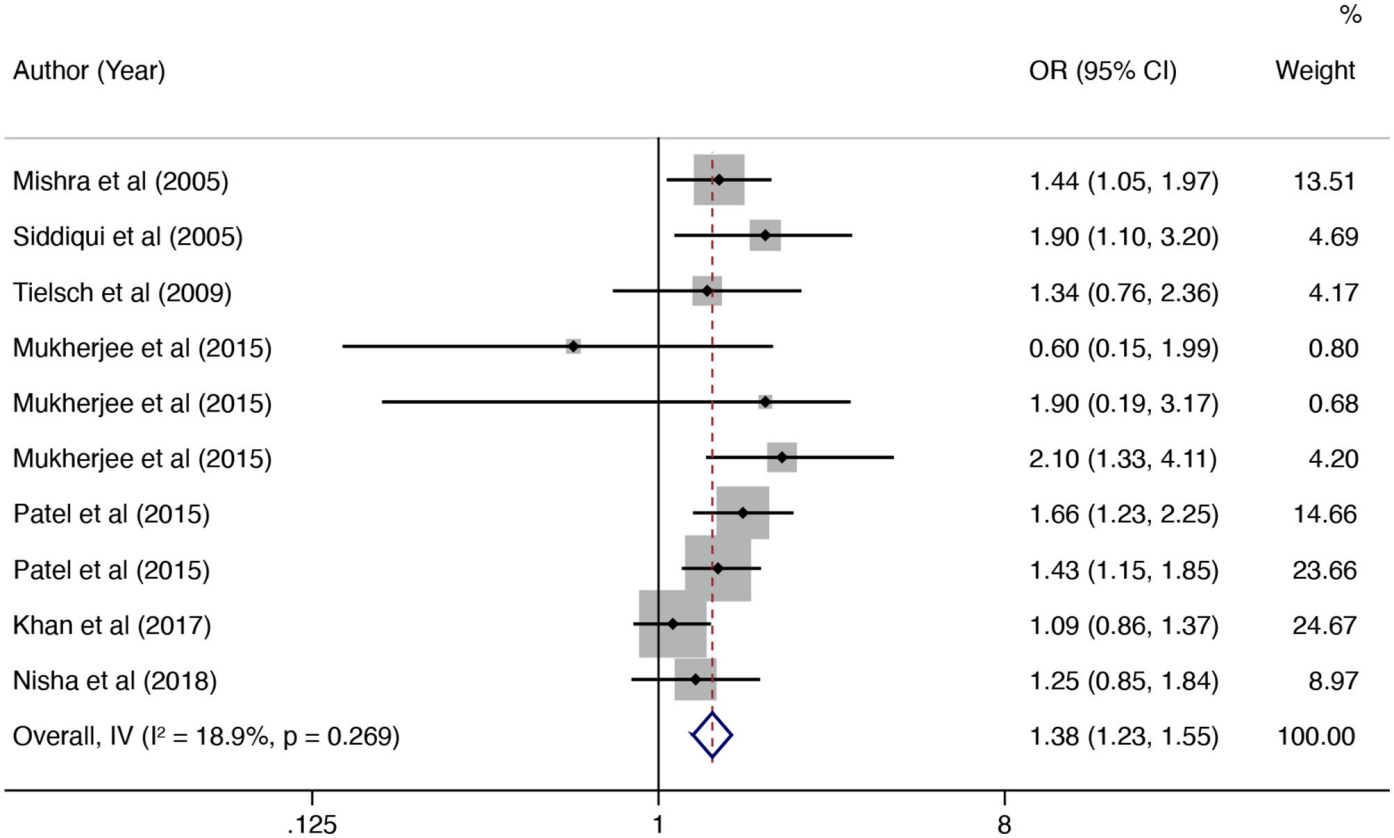
Forest plot showing the effect of polluting cooking fuels on stillbirth.

**Figure 5:**
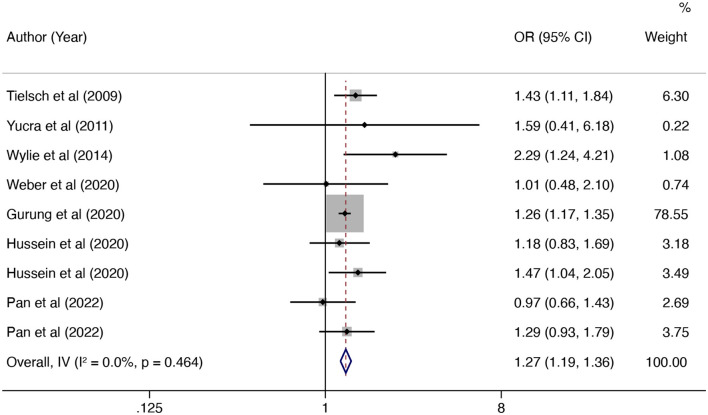
Forest plot showing the effect of polluting cooking fuels on preterm birth (PTB).

In this updated meta-analysis, we found polluting cooking fuels to be associated with LBW, SGA, stillbirth, and PTB. A total of 24 studies were included for investigating the relationship between polluting cooking fuels and LBW; the pooled OR was 1.37 (95% CI: 1.24, 1.52) with high heterogeneity (*I*^2^ = 75.0%, *P* < 0.001) (Figure 2). We found evidence of publication bias (Egger's test *P* < 0.001) (Supplementary Figure 1). After applying the trim and fill method, the corrected OR was 1.13 (95% CI: 1.01, 1.26). A total of 11 studies were explored for SGA; the pooled OR was 1.48 (95% CI: 1.13, 1.94), with high heterogeneity (*I*^2^ = 88.7%, *P* < 0.001) (Figure 3). No significant publication bias was found (Egger's test *P* = 0.468) (Supplementary Figure 2). For stillbirth, seven studies were explored, and the pooled OR was 1.38 (95% CI, 1.23, 1.55), with low statistical heterogeneity (*I*^2^ = 18.9%, *P* = 0.269) (Figure 4). We found no evidence of publication bias (Egger's test *P* = 0.584) (Supplementary Figure 3). Seven studies were explored for PTB; the pooled OR was 1.27 (95% CI: 1.19, 1.36), with low statistical heterogeneity (*I*^2^ = 0.0%, *P* = 0.464) (Figure 5). No evidence of publication bias was observed (Egger's test *P* = 0.568) (Supplementary Figure 4).

### Subgroup and meta-regression analyses

To explore possible sources of heterogeneity across eligible studies, we performed subgroup analyses for LBW and SGA (Table 2). Notably, the association between polluting cooking fuels and the risk of LBW was consistent in all subgroups examined, except for the subgroup conducted according to fuel types. In the subgroup analysis of cooking fuel type, biomass (OR: 1.57; 95% CI: 1.34–1.84) and fossil fuels (OR: 1.49; 95% CI: 1.10–2.02), but not mixed fuels (OR: 1.14; 95% CI: 0.98–1.32), were associated with LBW. Subgroup analysis based on location, the association between polluting cooking fuels, and risk of SGA was significant in Asia (OR: 1.47; 95% CI: 1.03–2.10), not in America (OR: 1.37; 95% CI: 0.87–2.18) or Africa (OR: 1.61; 95% CI: 0.79–3.28). In the subgroup analysis of study design, the association was significant in cohort studies (OR: 1.21; 95% CI: 1.13–1.30) not in cross-sectional studies (OR: 1.77; 95% CI: 0.73–4.32). Among cooking fuel types, both biomass (OR: 1.22; 95% CI: 1.05–1.42) and fossil fuels (OR: 1.29; 95% CI: 1.05–1.59), but not mixed fuels (OR: 2.40; 95% CI: 0.84–6.88), were associated with SGA.

**Table 2:** Pooled estimates for the association of polluting cooking fuels with LBW and SGA stratified according to the study characteristics.

	**No. of studies**	**Summary OR (95% CI)**	***I^2^* statistic (%)**	***P*-value**
**LBW**				
**Location**				
Asia	18	1.31 (1.19, 1.45)	75.3	< 0.001
America	1	3.73 (1.14, 12.15)	–	–
Africa	5	2.10 (1.24, 3.54)	69.3	0.002
**Study design**				
Cohort	9	1.33 (1.16, 1.54)	27.6	0.166
Case-control	4	3.51 (1.81, 6.80)	65.7	0.012
Cross-sectional	11	1.27 (1.13, 1.42)	78.1	< 0.001
**Sample size**				
< 1,000	11	2.03 (1.50, 2.73)	60.0	0.001
≥1,000	13	1.22 (1.12, 1.34)	74.1	< 0.001
**Cooking fuel type**				
Biomass	16	1.57 (1.34, 1.84)	72.6	< 0.001
Fossil	6	1.49 (1.10, 2.02)	58.7	0.024
Mixed	8	1.14 (0.98, 1.32)	78.7	< 0.001
**Outcome assessment**				
Direct	14	1.61 (1.34, 1.94)	53.9	0.002
Indirect	10	1.23 (1.10, 1.38)	78.7	< 0.001
**SGA**				
**Location**				
Asia	7	1.47 (1.03, 2.10)	93.6	< 0.001
America	2	1.37 (0.87, 2.18)	58.7	0.064
Africa	2	1.61 (0.79, 3.28)	0.0	0.974
**Study design**				
Cohort	6	1.21 (1.13, 1.30)	0.0	0.948
Case-control	1	4.53 (1.33, 15.46)	**–**	**–**
Cross-sectional	4	1.77 (0.73, 4.32)	95.2	< 0.001
**Sample size**				
< 1,000	4	1.97 (1.29, 3.03)	0.0	0.708
≥1,000	7	1.38 (1.01, 1.87)	92.1	< 0.001
**Cooking fuel type**				
Biomass	8	1.22 (1.05, 1.42)	28.0	0.178
Fossil	2	1.29 (1.05, 1.59)	0.0	0.916
Mixed	3	2.40 (0.84, 6.88)	95.7	< 0.001
**Outcome assessment**				
Direct	10	1.48 (1.12, 1.95)	89.4	< 0.001
Indirect	1	1.43 (0.41, 5.00)	–	–

OR, odds ratio; CI, confidence interval; LBW, low birth weight; SGA, small for gestational age.

In the meta-regression analysis of LBW (Supplementary Table 6), we found that study design (cohort study: *P* < 0.01; cross-sectional study: *P* < 0.01) and sample size (≥1,000: *P* < 0.01) were the covariates associated with heterogeneity. Cooking fuel types (mixed fuel: *P* < 0.05) were covariates associated with the heterogeneity in the SGA analysis.

### Sensitivity analysis

Sensitivity analysis results showed that excluding one study at a time did not significantly alter the overall effect of polluting fuel use on LBW (OR altered between 1.33 and 1.41) (Supplementary Table 7) and SGA (OR altered between 1.26 and 1.56) (Supplementary Table 8). Regarding the studies reporting SGA, heterogeneity was greatly reduced when Chaudhary's study (34) was excluded (*I*^2^ reduced to 15.9%) (Supplementary Table 8).

## Discussion

In a previous meta-analysis, Pope et al. reported that indoor air pollution from solid fuel use was associated with a 38% increased risk of LBW and a 51% increased risk of stillbirth (25). Amegah et al. reported that solid fuel use was associated with a 35% increased risk of LBW, a 30% increased risk of PTB, and a 29% increased risk of stillbirth (26). These results are consistent with ours.

This is the first meta-analysis to summarize the available evidence relating to polluting cooking fuels and SGA, and we found a positive association between them. The combustion of polluting cooking fuels emits high levels of pollutants, such as particulate matter (PM) and nitrogen dioxide (NO_2_), which have been shown to be associated with SGA in several meta-analyses (74–76). We included 11 relevant studies exploring the effect of polluting cooking fuels on SGA; further eligible studies are needed to verify the association.

In the subgroup analysis, the summary OR varied between different locations for SGA. This may be attributed to the different cooking fuel choices in the studies. For example, the majority of women in South Asia use wood (49.1–89.7%), and most of the women in Africa use charcoal (85.4–93.5%), whereas women in Latin America mainly use liquefied petroleum gas (69.1–97.6%) (77). However, this study only included five studies in South Asia (11, 33–36), two studies in Africa (44, 45), and one study in Latin America (28) to detect the association between polluting fuels and SGA. Therefore, more studies are needed to explore regional differences.

Different fuel types may influence the strength of the association between polluting fuels with LBW and SGA. The results demonstrated that biomass fuels had a larger pooled OR for the association with LBW than fossil fuels. However, when various polluting cooking fuels were grouped together, the association was not significant. Studies have shown that the concentrations of pollutants released from coal are lower than those released from biomass (78, 79). A laboratory assessment has also shown that the concentrations of pollutants released from fossil fuels (including kerosene) are lower than those from biomass (80). Therefore, the pooled OR could be higher between biomass fuels and LBW. However, we found the pooled OR of the association between fossil fuels and SGA was higher than that with biomass fuels. The result was consistent with the included studies (24, 37). In addition, the results of the included studies also showed higher OR values between biomass and LBW than fossil fuels. The two cohort studies were both conducted in China, and more studies are needed to confirm this conclusion and explore the potential biological mechanism. Studies have reported that the concentrations of pollutants are reduced when people use mixed fuels (78, 81). This could be attributed to the occasional use of relatively low polluting fuels (e.g., kerosene) (81), which masked the relationship between one type of polluting cooking fuel (biomass or fossil fuels) and adverse birth outcomes.

There was high heterogeneity in the present study, although the meta-regression analyses found several covariates associated with heterogeneity, which was still uncontrolled after subgroup analyses. Moreover, sensitivity analysis was performed to explore the potential source of heterogeneity in LBW and SGA. However, the source of heterogeneity was not found in LBW. This may be attributed to the number of included studies that were not sufficient to detect the source of heterogeneity. In the future, more qualified original research is needed to explore the source of heterogeneity. For SGA, sensitivity analysis revealed that after excluding Chaudhary's study (34), the heterogeneity was reduced greatly. In this cross-sectional study, mothers of SGA infants had higher rates of exposure to polluting fuels during pregnancy, and mothers with appropriate size for gestational age infants had lower rates of exposure. Therefore, the population with different exposure rates may be a source of heterogeneity. Furthermore, the adjustment for different covariates may be a source of heterogeneity. Compared with other studies, Chaudhary's study had additionally adjusted high carbohydrate snacks, pregnancy-induced hypertension, gestational diabetes, cardiovascular diseases, polyhydramnios, hypothyroid, and anemia.

Combustion of polluting cooking fuels emits high levels of pollutants, such as PM, CO, NO_2_, sulfur dioxide (SO_2_), and PAHs (37). Exposure to PM induces maternal systemic and placental oxidative stress and inflammation (82), which could result in suboptimal placentation, and subsequent fetal growth restriction (83). It is known that PAHs are linked to developmental and reproductive toxicity (84). They can result in inadequate transplacental nutrient exchange (85). Carbon monoxide binds to hemoglobin to form carboxyhemoglobin (86), which leads to inadequate oxygen supply to the fetus and hence fetal growth retardation (35). Moreover, it may cross the placental barrier, where it can act directly to affect fetal health and development (44). In short, both PM and toxic chemicals affect the growth and development of the fetus through various mechanisms, leading to adverse birth outcomes.

The current study had some strengths. In this updated meta-analysis, we provided the first quantitative assessment of the association between polluting cooking fuels and SGA. Compared with the previous study (26), this meta-analysis included 23 new studies and conducted subgroup and meta-regression analyses for LBW and SGA. In addition, we explored whether different types of polluting fuels use had a different impact on the risk of adverse birth outcomes.

### Study limitations and future perspectives

Our study also had some limitations. First, because of the observational nature of included studies, recall and selection bias cannot be eliminated. Although cohort studies are less vulnerable to such bias, only 11 cohort studies were included in the final analysis. In addition, cross-sectional and case–control studies have difficulties in determining the temporality between exposure and outcome. Consequently, our current results should be interpreted cautiously, and more prospective studies are needed to thoroughly investigate the association between polluting cooking fuels and adverse birth outcomes. Second, there is a possibility of misclassification of exposure and outcome assessment. All included studies collected information regarding primary cooking fuels through interviews. However, there is considerable variability in exposure, such as multiple fuel use and temporal changes in fuel use. Using only qualitative indicators (such as reported cooking fuel use) could produce considerable exposure misclassification for exposure settings and inaccurate correlation assessments between exposure and outcomes. By directly measuring and recording the concentration of kitchen air pollutants, we can avoid misclassification of exposures and obtain accurate exposure–response relationships. However, only three of included studies directly measured the concentration of kitchen air pollutants (28, 38, 40). It is important for future studies to employ more accurate methods, such as household or portable air quality monitors, to assess household air pollutant exposure. Moreover, the baby's birth size was used as a proxy for birth weight when the information could not be retrieved from a health card or maternal recall. However, previous findings demonstrated that the demographic and health survey data on birth size could be used as an alternative to birth weight (23). Third, although some potential confounding factors were taken into account in the original studies, other confounding factors, such as the type of cooking stoves, personal exposure, and the availability of windows or chimneys, still remained uncontrolled in some studies. A study conducted in Indonesia demonstrated that pollutant concentrations remained high even when the houses were adequately ventilated (31); however, the residual confounding effects of other important factors could not be eliminated, which might cause inaccurate evaluations of their effects on the risk of adverse birth outcomes. Finally, there was considerable heterogeneity in the current study. After conducting subgroup and meta-regression analyses, the heterogeneity was still uncontrolled. Moreover, sensitivity analysis suggested that no individual study significantly affected the pooled effect size. Therefore, the heterogeneity is the cause for caution concerning the conclusion. Strong epidemiological evidence estimating health risks from particular fuels and technologies utilized, and improved and standardized data collection capturing the fuels and technologies used in the home for all key end-uses, such as cooking, heating, and lighting, are needed. The understanding of exposure to total household air pollution will be improved through increased monitoring efforts combined with future modeling that takes stove stacking into account. This will better inform policy and programmatic decision-making as well as the global monitoring of health and environmental impacts.

## Conclusion

Our findings suggest that polluting cooking fuel use is associated with an increased risk of LBW, SGA, stillbirth, and PTB. Different polluting fuel types may influence the strength of the association between polluting fuel use and LBW and SGA. Moving forward, we encourage more prospective studies to thoroughly investigate the association between polluting cooking fuel use and adverse birth outcomes. On exposure assessment, a more direct method of measuring, such as household or portable air quality monitors, will help quantify exposure information and an exposure–response relationship.

## Data availability statement

The original contributions presented in the study are included in the article/Supplementary material, further inquiries can be directed to the corresponding authors.

## Author contributions

ML: conceptualization, data curation, software, formal analysis, and writing—original draft. TL, CM, JF, ZZ, and YW: writing—reviewing and editing. YX: writing—reviewing and editing and supervising. YZ: writing—reviewing and editing and funding acquisition. CJ: conceptualization, writing—reviewing and editing, supervision, and funding acquisition. All authors contributed to the article and approved the submitted version.
