# Supplementary material for: Household polluting cooking fuels and adverse birth outcomes: An updated systematic review and meta-analysis
Source: Front Public Health. 2023 Mar 3;11:978556. doi: 10.3389/fpubh.2023.978556 (PMC10020710; doi:10.3389/fpubh.2023.978556)
Supplement: Supplementary file 1 [file Data_Sheet_1.docx]

Table S1. Literature search strategy (dated to January 16, 2023).

| Databases | Search strategies | Results |
| --- | --- | --- |
| PubMed | ("air pollution, indoor"[MeSH Terms] OR "indoor air pollution"[Title/Abstract] OR "indoor air quality"[Title/Abstract] OR "household air pollution"[Title/Abstract] OR "household fuel"[Title/Abstract] OR "domestic fuel"[Title/Abstract] OR "cooking fuel"[Title/Abstract] OR "polluted fuel"[Title/Abstract] OR "unclean fuel"[Title/Abstract] OR "solid fuel"[Title/Abstract] OR "biofuels"[MeSH Terms] OR "biofuel"[Title/Abstract] OR "biomass fuel"[Title/Abstract] OR "biomass"[MeSH Terms] OR "biomasses"[Title/Abstract] OR "wood"[MeSH Terms] OR "woods"[Title/Abstract] OR "firewood"[Title/Abstract] OR "charcoal"[MeSH Terms] OR "crop residue"[Title/Abstract] OR "animal dung"[Title/Abstract] OR "coal"[MeSH Terms] OR "coals"[Title/Abstract] OR "kerosene"[MeSH Terms] OR "kerosine"[Title/Abstract] OR "cooking"[MeSH Terms] OR "cookery"[Title/Abstract] OR "biomass smoke"[Title/Abstract] OR "wood smoke"[Title/Abstract] OR "charcoal smoke"[Title/Abstract] OR "soot"[MeSH Terms] OR "cooking smoke"[Title/Abstract]) AND ("pregnancy outcome"[MeSH Terms] OR "pregnancy outcomes"[Title/Abstract] OR "pregnancy complications"[MeSH Terms] OR "pregnancy complication" [Title/Abstract] OR "adverse birth outcomes"[Title/Abstract] OR "adverse birth outcome"[Title/Abstract] OR "infant, low birth weight"[MeSH Terms] OR "low birth weight infant"[Title/Abstract] OR "low birth weight"[Title/Abstract] OR "low birth weights"[Title/Abstract] OR "premature birth"[MeSH Terms] OR "premature births"[Title/Abstract] OR "preterm birth"[Title/Abstract] OR "preterm births"[Title/Abstract] OR "stillbirth"[MeSH Terms] OR "stillbirths"[Title/Abstract] OR "infant, small for gestational age"[MeSH Terms]) | 441 |
| Web of Science | (TS=(air pollution, indoor or indoor air pollution or indoor air quality)) OR TS=(household air pollution)) OR TS=(household fuel)) OR TS=(domestic fuel)) OR TS=(cooking fuel)) OR TS=(polluted fuel)) OR TS=(unclean fuel)) OR TS=(solid fuel)) OR TS=(biofuels or biofuel)) OR TS=(biomass fuel)) OR TS=(biomass or biomasses)) OR TS=(wood or woods)) OR TS=(firewood)) OR TS=(charcoal)) OR TS=(crop residue)) OR TS=(animal dung)) OR TS=(coal or coals)) OR TS=(kerosene or kerosine)) OR TS=(cooking or cookery)) OR TS=(biomass smoke)) OR TS=(wood smoke)) OR TS=(charcoal smoke)) OR TS=(soot)) OR TS=(cooking smoke)) AND (TS=(pregnancy outcome or pregnancy outcomes)) OR TS=(pregnancy complications or pregnancy complication or adverse birth outcomes or adverse birth outcome)) OR TS=(infant, low birth weight or low birth weight infant or low birth weight or low birth weights)) OR TS=(premature birth or premature births or preterm birth or preterm births)) OR TS=(stillbirth or stillbirths)) OR TS=(infant, small for gestational age)) | 943 |
| Embase | ('indoor air pollution'/exp OR 'household air pollution':ab,ti OR 'household fuel':ab,ti OR 'domestic fuel':ab,ti OR 'cooking fuel':ab,ti OR 'polluted fuel':ab,ti OR 'unclean fuel':ab,ti OR 'solid fuel':ab,ti OR 'biofuel'/exp OR biofuels:ab,ti OR 'biomass fuel':ab,ti OR 'biomass'/exp OR 'wood'/exp OR firewood:ab,ti OR 'charcoal'/exp OR 'crop residue':ab,ti OR 'animal dung':ab,ti OR 'coal'/exp OR 'kerosene'/exp OR kerosine:ab,ti OR 'cooking'/exp OR cookery:ab,ti OR 'biomass smoke':ab,ti OR 'wood smoke':ab,ti OR 'charcoal smoke':ab,ti OR 'soot'/exp OR 'cooking smoke':ab,ti) AND ('pregnancy outcome'/exp OR 'birth outcome':ab,ti OR 'obstetric outcome':ab,ti OR 'adverse birth outcome':ab,ti OR 'low birth weight'/exp OR 'lbw infant':ab,ti OR 'lbw neonate':ab,ti OR 'lbw newborn':ab,ti OR 'low birth weight infant':ab,ti OR 'low birthweight':ab,ti OR 'premature labor'/exp OR 'premature delivery':ab,ti OR 'premature labour':ab,ti OR 'premature obstetric labor':ab,ti OR 'premature obstetric labour':ab,ti OR 'preterm birth':ab,ti OR 'preterm delivery':ab,ti OR 'preterm labor':ab,ti OR 'preterm labour':ab,ti OR 'stillbirth'/exp OR 'stillborn baby':ab,ti OR 'small for date infant'/exp OR 'sga infant':ab,ti OR 'sga neonate':ab,ti OR 'sga newborn':ab,ti OR 'small for age infant':ab,ti OR 'small for date baby':ab,ti OR 'small for gestational age':ab,ti OR 'small for gestational age infant':ab,ti) | 421 |
| Scopus | ( ( ( TITLE-ABS-KEY ( "air pollution, indoor" ) OR TITLE-ABS-KEY ( "indoor air pollution" ) OR TITLE-ABS-KEY ( "indoor air quality" ) ) ) OR ( TITLE-ABS-KEY ( "household air pollution" ) ) OR ( TITLE-ABS-KEY ( "household fuel" ) ) OR ( TITLE-ABS-KEY ( "domestic fuel" ) ) OR ( TITLE-ABS-KEY ( "cooking fuel" ) ) OR ( TITLE-ABS-KEY ( "polluted fuel" ) ) OR ( TITLE-ABS-KEY ( "unclean fuel" ) ) OR ( TITLE-ABS-KEY ( "solid fuel" ) ) OR ( ( TITLE-ABS-KEY ( biofuels ) OR TITLE-ABS-KEY ( biofuel ) ) ) OR ( TITLE-ABS-KEY ( "biomass fuel" ) ) OR ( ( TITLE-ABS-KEY ( biomass ) OR TITLE-ABS-KEY ( biomasses ) ) ) OR ( ( TITLE-ABS-KEY ( wood ) OR TITLE-ABS-KEY ( woods ) ) ) OR ( TITLE-ABS-KEY ( firewood ) ) OR ( TITLE-ABS-KEY ( charcoal ) ) OR ( TITLE-ABS-KEY ( "crop residue" ) ) OR ( TITLE-ABS-KEY ( "animal dung" ) ) OR ( ( TITLE-ABS-KEY ( coal ) OR TITLE-ABS-KEY ( coals ) ) ) OR ( ( TITLE-ABS-KEY ( kerosene ) OR TITLE-ABS-KEY ( kerosine ) ) ) OR ( ( TITLE-ABS-KEY ( cooking ) OR TITLE-ABS-KEY ( cookery ) ) ) OR ( TITLE-ABS-KEY ( "biomass smoke" ) ) OR ( TITLE-ABS-KEY ( "wood smoke" ) ) OR ( TITLE-ABS-KEY ( "charcoal smoke" ) ) OR ( TITLE-ABS-KEY ( soot ) ) OR ( TITLE-ABS-KEY ( "cooking smoke" ) ) ) AND ( ( ( TITLE-ABS-KEY ( "pregnancy outcome" ) OR TITLE-ABS-KEY ( "pregnancy outcomes" ) ) ) OR ( ( TITLE-ABS-KEY ( "pregnancy complications" ) OR TITLE-ABS-KEY ( "pregnancy complication" ) OR TITLE-ABS-KEY ( "adverse birth outcomes" ) OR TITLE-ABS-KEY ( "adverse birth outcome" ) ) ) OR ( ( TITLE-ABS-KEY ( "infant, low birth weight" ) OR TITLE-ABS-KEY ( "low birth weight infant" ) OR TITLE-ABS-KEY ( "low birth weight" ) OR TITLE-ABS-KEY ( "low birth weights" ) ) ) OR ( ( TITLE-ABS-KEY ( "premature birth" ) OR TITLE-ABS-KEY ( "premature births" ) OR TITLE-ABS-KEY ( "preterm birth" ) OR TITLE-ABS-KEY ( "preterm births" ) ) ) OR ( ( TITLE-ABS-KEY ( stillbirth ) OR TITLE-ABS-KEY ( stillbirths ) ) ) OR ( TITLE-ABS-KEY ( "infant, small for gestational age" ) ) ) | 798 |

Table S2. Association between polluting cooking fuels and adverse birth outcomes.

| **Author** | **Year** | **Exposed group [n (% ^a^)]** | **Exposure assessment** | **Comparator group** | **Outcomes [n (% ^b^)]** | **Outcome Assessment** | **Results** |
| --- | --- | --- | --- | --- | --- | --- | --- |
| Mishra et al | 2005 | Biomass: 9,877 (53.2) | Interview | Cleaner fuels (electricity, liquid petroleum gas, biogas, or kerosene) | Stillbirth biomass: 888 (9.0); cleaner fuels: 143 (4.0) | NFHS maternal recall | Stillbirth aOR: 1.44 (95% CI: 1.05, 1.97) |
| Siddiqui et al | 2005 | Wood ^c^ | Interview | Natural gas | LBW Stillbirth | Health card | LBW aOR: 1.77 (95% CI: 1.20, 2.50) Stillbirth aOR: 1.90 (95% CI: 1.10, 3.20) |
| Siddiqui et al | 2008 | Wood: 366 (57.7) | Interview | Natural gas | LBW wood: 83 (22.7); natural gas: 40 (15.0) | Records | LBW aOR: 1.86 (95% CI: 1.11, 3.14) |
| Tielsch et al | 2009 | Wood or Dung ^c^ | Interview | No wood or dung | LBW SGA PTB Stillbirth | Birth weight was measured using an electronic digital infant scale | LBW aRR: 1.49 (95% CI: 1.25, 1.77) SGA aRR: 1.21 (95% CI: 1.11, 1.31) PTB aRR: 1.43 (95% CI: 1.11, 1.84) Stillbirth aRR: 1.34 (95% CI: 0.76, 2.36) |
| Sreeramareddy et al | 2011 | High pollution fuels: 34,365 (72.9) | Interview | Low pollution fuels (electricity, liquid petroleum gas, natural gas and biogas) | LBW high pollution fuels:7,634 (22.2); low pollution fuels: 2,016 (15.8) | Child health card and/or mothers’ recall | LBW aOR: 1.07 (95% CI: 0.94, 1.22) |
| Yucra et al | 2011 | Biofuel: 77 (40.5) | Interview | Gas | LBW  PTB | Hospital records | LBW aOR: 3.73 (95% CI: 1.14, 12.1) PTB aOR: 1.59 (95% CI: 0.41, 6.18) |
| Abusalah et al | 2012 | Wood: 79 (17.7) | Interview | Not exposed to wood | LBW wood: 55 (69.6); not exposed to wood: 168 (45.8) | Birth weight was measured by trained qualified nurses | LBW aOR: 2.3 (95% CI:1.2, 4.7) |
| Amegah et al | 2012 | Charcoal: 282 (47.6) | Interview | Liquefied petroleum gas (LPG) | LBW charcoal: 64 (22.7); LPG: 15 (9.3) | Hospital records | LBW aRR: 1.41 (95% CI: 0.62, 3.23) |
| Epstein et al | 2013 | Biomass: 6,195 (41.7); Kerosene: 855(5.8); Coal: 286 (1.9) | Interview | Gas | LBW biomass: 1,412 (22.8); kerosene: 167 (19.6); coal: 67 (23.6); gas: 1,239 (16.5) | India’s National Family Health Survey (NFHS-3) | LBW aOR: biomass: 1.24 (95% CI: 1.04, 1.48); kerosene: 1.51 (95% CI: 1.08, 2.12); coal: 1.57 (95% CI: 1.03, 2.41) |
| Wylie et al | 2014 | Wood: 1,306 (74.9) | Interview | Gas | LBW wood: 286 (21.9); gas: 48 (18.1) SGA wood:71 (5.4); gas: 20 (7.5) PTB wood: 390 (29.8); gas:33 (12.5) | All neonates were weighed with an electronic digital scale to the nearest 10 grams. The gestational ages of all live births were estimated within 24 hours of delivery by means of a modified Ballard examination | LBW aOR: 0.95 (95% CI: 0.58, 1.57) SGA aOR: 0.53 (95% CI: 0.23, 1.19) PTB aOR: 2.29 (95% CI: 1.24, 4.21) |
| Yucra et al | 2014 | Biofuel: 75 (37.1) | Interview | Gas | SGA biofuel: 43 (57.3); gas: 30 (43.5) | Medical records | SGA aOR: 4.53 (95% CI: 1.33, 15.49) |
| Demelash et al | 2015 | Firewood: 272 (70.3); Kerosene: 24 (6.2); Animal dung:32 (8.3) | Interview | Electricity | LBW firewood: 82 (30.1); kerosene: 13 (54.2); animal dung: 23 (71.9); electricity: 5 (10.9) | The weight of the newborns was measured within 15 min after birth using a balanced Seca scale | LBW aOR: firewood: 2.70 (95%CI: 1.00, 7.17); kerosene: 8.90 (95%CI: 2.54, 31.11); animal dung: 14.4 (95%CI: 4.08, 50.97) |
| Jiang et al | 2015 | Coal: 358 (3.6); Biomass: 120 (1.2) | Interview | Gas | LBW coal: 70 (19.6); biomass: 42 (35.0); gas: 371 (4.7) SGA coal: 55 (15.4); biomass: 22 (18.3); gas: 509 (6.4) | Medical records | LBW aOR: coal: 1.09 (95% CI: 0.67, 1.78); biomass: 2.51 (95% CI: 1.26, 5.01) SGA aOR: coal: 1.27 (95% CI: 0.90, 1.80), biomass: 1.22 (95% CI 0.70, 2.08) |
| Mukherjee et al | 2015 | Biomass: 227 (56.2) | Interview | LPG | LBW biomass: 67 (29.5); LPG: 12 (6.8) Stillbirth biomass: 22 (9.7); LPG: 6 (3.4) | Maternal recall | LBW aOR: lifetime exposure of 5,000-10,000 h: 0.7 (95% CI: 0.2, 2.45); >10,000–15,000 h: 1.9 (95% CI: 1.18, 5.21); >15,000 h: 5.1 (95% CI: 2.06, 7.34) Stillbirth aOR: 5,000-10,000 h: 0.6 (95% CI: 0.15, 1.99); >10,000–15,000 h: 1.9 (95% CI: 0.19, 3.17); >15,000 h: 2.1 (95% CI: 1.33, 4.11) |
| Patel et al | 2015 | Polluting fuel: 54,082 (82.1) | Interview | Clean fuel. (electricity, biogas, LPG, natural gas) | Macerated stillbirth  non-macerated stillbirth | Interview, hospital records | Macerated stillbirth aOR: 1.66 (95% CI: 1.23, 2.25) non-macerated stillbirth aOR: 1.43 (95% CI: 1.15, 1.85) |
| Haider et al | 2016 | Coal; Wood; Straw/Crop ^c^ | 2011 BDHS | Electricity/Gas | LBW | BDHS maternal recall of baby size | LBW aOR: coal: 2.6 (95% CI: 1.1, 6.2); wood: 1.1 (95% CI: 1.0, 1.2); straw/crop: 1.1 (95% CI: 1.0, 1.3) |
| Khan et al | 2017 | Solid fuel: 20,465 (89.8) | 2007, 2011, 2014 BDHS | Clean fuel (electricity, LPG, natural gas, biogas) | LBW solid: 2,140 (10.5); clean: 328 (14.1) Stillbirth solid: 458 (2.2); clean: 69 (3.0) | BDHS maternal recall | LBW aOR: 0.96 (95% CI: 0.81, 1.13) Stillbirth aOR: 1.09 (95% CI: 0.86, 1.37) |
| Balakrishnan et al | 2018 | Kerosene: 98 (8.7); Biomass: 171 (15.3) | Interview | LPG | LBW kerosene: 17 (17.3); biomass: 34 (19.9); LPG: 106 (14.7) | Medical records | LBW OR: kerosene: 1.22 (95% CI: 0.69, 2.13); biomass: 1.44 (95% CI: 0.94, 2.21) |
| Nisha et al | 2018 | Polluting fuel: 24,161 (88.7) | 2004, 2007, 2011, 2014 BDHS | Clean fuel (electricity, liquefied petroleum gas, natural gas, and biogas) | Stillbirth polluting: 703 (2.9); clean: 64 (2.1) | BDHS maternal recall | Stillbirth aOR: 1.25 (95% CI: 0.85, 1.84) |
| Suryadhi et al | 2019 | Solid fuel: 15,294 (41.6) | 2012 IDHS | Clean fuel (electricity, propane/natural gas, biogas, or kerosene) | LBW | IDHS maternal recall | LBW aOR: 1.61 (95% CI: 1.37, 1.89) |
| Fleisch et al | 2020 | Wood: 536 (43.8) | Interview | Did not use wood | SGA wood: 60 (11.2); did not use wood: 65 (9.5) | Records | SGA aOR: first trimester: 1.03 (95%CI: 0.67, 1.55); second trimester: 1.00 (95%CI: 0.63, 1.56); third trimester: 1.81 (95%CI: 0.96, 3.38) |
| Gurung et al | 2020 | Polluted fuel: 12,695 (25.3) | Interview | Clean fuel | PTB polluted: 1,384 (20.9); clean: 2,886 (7.7) | Records | PTB aOR: 1.26 (95%CI: 1.17, 1.35) |
| K et al | 2020 | Firewood and kerosene: 245 (66.4) | Interview | LPG and bio gas | LBW firewood and kerosene: 87 (35.5); LPG and bio gas: 36 (29.0) | Antenatal Care (ANC) card and Maternal and newborn register | LBW aOR: 1.4 (95% CI: 0.7, 2.6) |
| Gautam Paudel et al | 2020 | Polluted fuel: 14,913 (24.6) | Interview | Clean fuel | SGA polluted: 2,033 (13.6); clean: 2,721 (11.2) | Measuring gestational age by last menstrual period (LMP) and birth weight (grams) | SGA aOR: 1.51 (95% CI: 1.16, 1.97) |
| Weber et al | 2020 | Polluting fuel: 279 (34.1) | Interview | Clean fuel (LPG, electricity) | LBW polluting: 30 (10.8); clean: 56 (10.4) SGA polluting: 7 (2.5); clean: 11 (2.0) PTB polluting: 20 (7.2); clean: 34 (6.3) | Hospital records, maternal recall | LBW aOR: 1.05 (95% CI: 0.57, 1.93) SGA aOR: 1.43 (95% CI: 0.40, 4.89) PTB aOR: 1.01 (95% CI: 0.48, 2.10) |
| Hussein et al | 2020 | Firewood: 463 (35.0); Charcoal: 758 (57.3) | Interview | Gas/electricity/ biogas | LBW firewood: 38 (8.2); charcoal: 32 (4.2); gas: 4 (4.0) SGA firewood: 24 (5.2); charcoal: 26 (3.4); ga: 3 (3.0) PTB firewood: 147 (31.7); charcoal: 308 (40.6); gas: 27 (27.0) | Records | LBW aRR: firewood: 1.23 (95%CI: 0.41, 3.71); charcoal: 1.34 (95%CI: 0.45, 3.97) SGA aRR: firewood: 1.70 (95%CI: 0.49, 5.92); charcoal: 1.72 (95%CI: 0.52, 5.65) PTB aRR: firewood: 1.18 (95%CI: 0.83, 1.69); charcoal: 1.47 (95%CI: 1.04, 2.05) |
| Chaudhary et al | 2021 | Solid fuel: 1,226 (30.7) | Interview | LPG or electricity | SGA solid: 518 (42.3); LPG or electricity: 295 (10.6) | Gestational age was estimated by the first day of the LMP and ultrasonography record (where available) or New Ballard's scoring system. Birthweight was measured within 24 hours of birth using an electronic weighing machine. | SGA aOR: 5.4 (95% CI: 4.1, 6.9) |
| Islam et al | 2021 | Unclean cooking fuels: 60,932 (65.0) | 2015-2016 NFHS | Clean cooking fuels (electricity, liquefied petroleum gas (LPG), biogas) | LBW unclean: 10,602 (17.4); clean: 4,983 (15.2) | NFHS maternal recall, health card | LBW aOR: 1.03 (95% CI: 0.97, 1.08) |
| Kanno et al | 2021 | High- pollution cooking fuels: 9,703 (96.89) | 2016 EDHS | Low-pollution cooking fuels (electricity, liquid petroleum gas, natural gas and biogas) | LBW  high-pollution cooking fuels: 2,538 (26.16); low-pollution cooking fuels: 53 (17.04) | Health cards, mother's recall | LBW  aOR: 1.4 (95% CI: 0.98, 1.9) |
| Vakalopoulos et al | 2021 | Biomass fuel: 215 (48.3) | Interview | Clean fuel (LPG, biogas, electricity) | LBW biomass: 34 (15.8); clean: 24 (10.4) SGA biomass: 100 (46.5); clean: 79 (34.3) | Health card | LBW aOR: 2.74 (95% CI: 1.08, 6.96) SGA aOR: 1.87 (95% CI: 1.03, 3.41) |
| Lu et al | 2022 | Coal/wood: 2,327 (7.57) | Interview | Electricity | LBW | Recall | LBW  aOR: 0.82 (95% CI: 0.53, 1.28) |
| Pan et al | 2022 | Coal: 613 (5.9); Wood: 700 (6.8) | Interview | Gas | LBW coal: 35 (11.1); wood: 50 (7.1); gas: 409 (5.1) SGA coal: 82 (13.4); wood: 89 (12.7); gas: 787 (9.9) PTB coal: 31 (5.1); wood: 48 (6.9); gas: 406 (5.1) | Gestational age was determined by combining ultrasound examination and mother-reported last menstrual period | LBW aRR: coal: 1.04 (95%CI: 0.72, 1.51); wood: 1.24 (95%CI: 0.90, 1.70) SGA aRR: coal: 1.30 (95%CI: 1.00, 1.68); wood: 1.16 (95%CI: 0.90, 1.49) PTB aRR: coal: 0.97 (95%CI: 0.66, 1.43); wood: 1.29 (95%CI: 0.93, 1.79) |

Note: OR: odds ratio; RR: relative risk; CI: confidence interval; LBW: low birth weight; PTB: preterm birth; SGA: small for gestational age.

^a^ The percentage is obtained by dividing the number of exposed subjects by the total number of participants.

^b^ The percentage is obtained by dividing the number of events by the total number of exposed or not exposed subjects.

^C^ The percentage cannot get from the study.

Table S3. Quality assessment of cohort studies.

|  | **Selection** | | | | **Comparability** | **Outcome** | | | **Total score** |
| --- | --- | --- | --- | --- | --- | --- | --- | --- | --- |
|  | Representative of the exposed cohort | Selection of the non-exposed cohort | Ascertainment of exposure | Outcome of interest not present at the start of study | Control for maternal age or additional factor | Assessment of outcome | Follow-up till delivery | Adequacy of follow-up of cohorts |  |
| Siddiqui et al (2005) | ❊ | ❊ | ❊ | ❊ | ❊ | ❊ | ❊ | - | 7 |
| Siddiqui et al (2008) | ❊ | ❊ | ❊ | - | ❊❊ | ❊ | - | - | 6 |
| Tielsch et al (2009) | ❊ | ❊ | ❊ | ❊ | ❊❊ | ❊ | ❊ | ❊ | 9 |
| Jiang et al (2015) | ❊ | ❊ | ❊ | - | ❊❊ | ❊ | - | - | 6 |
| Patel et al (2015) | ❊ | ❊ | ❊ | ❊ | ❊ | ❊ | ❊ | - | 7 |
| Balakrishnan et al (2018) | ❊ | ❊ | ❊ | ❊ | - | ❊ | ❊ | ❊ | 7 |
| Fleisch et al (2020) | ❊ | ❊ | ❊ | - | ❊❊ | ❊ | - | - | 6 |
| Weber et al (2020) | ❊ | ❊ | ❊ | - | ❊❊ | ❊ | - | - | 6 |
| Hussein et al (2020) | ❊ | ❊ | ❊ | ❊ | ❊ | ❊ | ❊ | ❊ | 8 |
| Lu et al  (2022) | ❊ | ❊ | ❊ | - | ❊❊ | - | - | - | 5 |
| Pan et al  (2022) | ❊ | ❊ | ❊ | ❊ | ❊❊ | ❊ | ❊ | - | 8 |

Table S4. Quality assessment of case-control studies.

|  | **Selection** | | | | **Comparability** | **Exposure** | | | **Total Score** |
| --- | --- | --- | --- | --- | --- | --- | --- | --- | --- |
|  | Adequate case definition | Representative of cases | Selection of controls | Definition of controls | Control for maternal age or additional factor | Ascertainment of exposure | Same method of ascertainment for participants | Nonresponse rate |  |
| Yucra et al (2011) | ❊ | - | - | ❊ | ❊❊ | ❊ | ❊ | - | 6 |
| Abusalah et al (2012) | ❊ | - | - | ❊ | ❊❊ | ❊ | ❊ | - | 6 |
| Yucra et al (2014) | ❊ | - | - | ❊ | ❊❊ | ❊ | ❊ | - | 6 |
| Demelash et al (2015) | ❊ | ❊ | - | ❊ | - | ❊ | ❊ | ❊ | 6 |
| K et al (2020) | ❊ | ❊ | - | ❊ | - | ❊ | ❊ | - | 5 |

Table S5. Quality assessment of cross-sectional studies.

|  | Define the source of information (survey, record review) | List inclusion and exclusion criteria for exposed and unexposed subjects (cases and controls) or refer to previous publications | Indicate time period used for identifying patients | Indicate whether or not subjects were consecutive, if not population -based | Indicate if evaluators of subjective components of study were masked to other aspects of the status of the participants | Describe any assessment undertaken for quality assurance purposes (e.g., test/retest of primary outcome measurements) | Explain any patient exclusion from analysis | Describe how confounding factors were assessed and/or controlled | If applicable, explain how missing data were handled in the analysis | Summarize patient response rates and completeness of data collection | Clarify what follow-up, if any, was expected and the percentage of patients for which incomplete data or follow-up were obtained | Total score |
| --- | --- | --- | --- | --- | --- | --- | --- | --- | --- | --- | --- | --- |
| Mishra et al (2005) | 1 | 1 | 1 | 0 | 0 | 0 | 1 | 1 | 0 | 1 | 0 | 6 |
| Sreeramareddy et al (2011) | 1 | 1 | 1 | 0 | 0 | 0 | 0 | 1 | 1 | 1 | 0 | 6 |
| Amegah et al (2012) | 1 | 1 | 1 | 0 | 0 | 1 | 0 | 1 | 0 | 1 | 0 | 6 |
| Epstein et al (2013) | 1 | 1 | 1 | 0 | 0 | 0 | 1 | 1 | 0 | 0 | 0 | 5 |
| Wylie et al (2014) | 1 | 1 | 1 | 0 | 0 | 1 | 0 | 1 | 0 | 0 | 0 | 5 |
| Mukherjee et al (2015) | 1 | 1 | 0 | 0 | 0 | 0 | 1 | 1 | 0 | 0 | 0 | 4 |
| Haider et al (2016) | 1 | 1 | 1 | 0 | 0 | 0 | 0 | 1 | 0 | 0 | 0 | 4 |
| Khan et al (2017) | 1 | 1 | 1 | 0 | 0 | 0 | 1 | 1 | 0 | 1 | 0 | 6 |
| Nisha et al (2018) | 1 | 1 | 1 | 0 | 0 | 0 | 0 | 1 | 0 | 1 | 0 | 5 |
| Suryadhi et al (2019) | 1 | 1 | 1 | 0 | 0 | 0 | 0 | 1 | 0 | 1 | 0 | 5 |
| Gurung et al (2020) | 1 | 1 | 1 | 0 | 0 | 0 | 0 | 1 | 1 | 0 | 0 | 5 |
| Gautam Paudel et al (2020) | 1 | 1 | 1 | 0 | 0 | 1 | 0 | 1 | 0 | 0 | 0 | 5 |
| Chaudhary et al (2021) | 1 | 1 | 1 | 0 | 0 | 1 | 0 | 1 | 0 | 0 | 0 | 5 |
| Islam et al (2021) | 1 | 1 | 1 | 0 | 0 | 0 | 1 | 1 | 0 | 0 | 0 | 5 |
| Kanno et al  (2021) | 1 | 1 | 0 | 0 | 0 | 0 | 1 | 1 | 0 | 0 | 0 | 4 |
| Vakalopoulos et al (2021) | 1 | 1 | 1 | 0 | 0 | 0 | 0 | 1 | 0 | 1 | 0 | 5 |

Table S6. Meta-regression analysis of low birth weight (LBW) and small for gestational age (SGA).

| **logor** | **Coef.** | **Std. Err.** | **t** | **P>\|t\|** | **[95% Conf. Interval]** |
| --- | --- | --- | --- | --- | --- |
| **LBW** |  |  |  |  |  |
| Location |  |  |  |  |  |
| Africa (ref) |  |  |  |  |  |
| America | 0.71 | 0.81 | 0.88 | 0.385 | -0.93, 2.36 |
| Asia | -0.30 | 0.22 | -1.35 | 0.186 | -0.76, 0.15 |
| Study design |  |  |  |  |  |
| Case-control study (ref) |  |  |  |  |  |
| Cohort study | -0.81 | 0.27 | -3.02 | 0.005 | -1.36, -0.27 |
| Cross-sectional study | -0.82 | 0.26 | -3.16 | 0.003 | -1.35, -0.29 |
| Sample size |  |  |  |  |  |
| < 1000 (ref) |  |  |  |  |  |
| ≥ 1000 | -0.44 | 0.15 | -3.00 | 0.005 | -0.74, -0.14 |
| Cooking fuels type |  |  |  |  |  |
| Biomass fuel (ref) |  |  |  |  |  |
| Fossil fuel | -0.08 | 0.20 | -0.38 | 0.708 | -0.48, 0.33 |
| Mixed fuel | -0.33 | 0.17 | -2.00 | 0.053 | -0.67, 0.01 |
| Outcome assessment |  |  |  |  |  |
| Direct (ref) |  |  |  |  |  |
| Indirect | -0.23 | 0.15 | -1.55 | 0.131 | -0.52, 0.07 |
| **SGA** |  |  |  |  |  |
| Location |  |  |  |  |  |
| Africa (ref) |  |  |  |  |  |
| America | -0.10 | 0.55 | -0.19 | 0.852 | -1.30, 1.09 |
| Asia | -0.09 | 0.50 | -0.18 | 0.857 | -1.16, 0.98 |
| Study design |  |  |  |  |  |
| Case-control study (ref) |  |  |  |  |  |
| Cohort study | -1.28 | 0.76 | -1.68 | 0.116 | -2.92, 0.36 |
| Cross-sectional study | -0.83 | 0.78 | -1.06 | 0.307 | -2.52, 0.86 |
| Sample size |  |  |  |  |  |
| < 1000 (ref) |  |  |  |  |  |
| ≥ 1000 | -0.38 | 0.36 | -1.06 | 0.305 | -1.15, 0.39 |
| Cooking fuels type |  |  |  |  |  |
| Biomass fuel (ref) |  |  |  |  |  |
| Fossil fuel | 0.00 | 0.33 | 0.01 | 0.994 | -0.71, 0.71 |
| Mixed fuel | 0.71 | 0.31 | 2.32 | 0.037 | 0.05, 1.38 |
| Outcome assessment |  |  |  |  |  |
| Direct (ref) |  |  |  |  |  |
| Indirect | -0.04 | 0.81 | -0.04 | 0.965 | -1.77, 1.70 |

Table S7. Sensitivity analysis with summary estimate with 95% confidence interval (CI) of low birth weight (LBW) from polluting cooking fuel exposure after removal of single study result (leave-one-out analysis).

| Study omitted | Estimate | Lower CI | Upper CI | *I*^2^ |
| --- | --- | --- | --- | --- |
| Siddiqui et al (2005) | 1.36 | 1.23 | 1.50 | 74.7 |
| Siddiqui et al (2008) | 1.36 | 1.23 | 1.51 | 75.1 |
| Tielsch et al (2009) | 1.36 | 1.23 | 1.51 | 73.9 |
| Sreeramareddy et al (2011) | 1.40 | 1.26 | 1.56 | 75.6 |
| Yucra et al (2011) | 1.36 | 1.23 | 1.51 | 75.0 |
| Abusalah et al (2012) | 1.36 | 1.23 | 1.50 | 75.0 |
| Amegah et al (2012) | 1.37 | 1.24 | 1.52 | 75.7 |
| Epstein et al (2013) | 1.38 | 1.24 | 1.54 | 75.6 |
| Epstein et al (2013) | 1.37 | 1.23 | 1.51 | 75.3 |
| Epstein et al (2013) | 1.37 | 1.23 | 1.52 | 75.2 |
| Wylie et al (2014) | 1.39 | 1.25 | 1.54 | 75.6 |
| Demelash et al (2015) | 1.36 | 1.23 | 1.51 | 75.2 |
| Demelash et al (2015) | 1.35 | 1.22 | 1.49 | 73.8 |
| Demelash et al (2015) | 1.34 | 1.22 | 1.48 | 72.7 |
| Jiang et al (2015) | 1.36 | 1.23 | 1.50 | 74.8 |
| Jiang et al (2015) | 1.38 | 1.25 | 1.53 | 75.7 |
| Mukherjee et al (2015) | 1.38 | 1.24 | 1.53 | 75.6 |
| Mukherjee et al (2015) | 1.36 | 1.23 | 1.51 | 75.4 |
| Mukherjee et al (2015) | 1.33 | 1.20 | 1.46 | 71.3 |
| Haider et al (2016) | 1.36 | 1.23 | 1.51 | 75.1 |
| Haider et al (2016) | 1.41 | 1.26 | 1.58 | 75.6 |
| Haider et al (2016) | 1.40 | 1.26 | 1.56 | 75.7 |
| Khan et al (2017) | 1.40 | 1.26 | 1.56 | 75.0 |
| Balakrishnan et al (2018) | 1.38 | 1.24 | 1.53 | 75.7 |
| Balakrishnan et al (2018) | 1.37 | 1.24 | 1.52 | 75.5 |
| Suryadhi et al (2019) | 1.35 | 1.22 | 1.49 | 72.0 |
| K et al (2020) | 1.37 | 1.24 | 1.52 | 75.7 |
| Weber et al (2020) | 1.38 | 1.25 | 1.53 | 75.7 |
| Hussein et al (2020) | 1.37 | 1.24 | 1.52 | 75.7 |
| Hussein et al (2020) | 1.37 | 1.24 | 1.52 | 75.7 |
| Islam et al (2021) | 1.41 | 1.26 | 1.58 | 71.0 |
| Kanno et al (2021) | 1.37 | 1.24 | 1.52 | 75.5 |
| Vakalopoulos et al (2021) | 1.36 | 1.23 | 1.51 | 75.1 |
| Lu et al (2022) | 1.39 | 1.26 | 1.54 | 75.4 |
| Pan et al (2022) | 1.39 | 1.25 | 1.54 | 75.7 |
| Pan et al (2022) | 1.38 | 1.24 | 1.53 | 75.7 |

Table S8. Sensitivity analysis with summary estimate with 95% confidence interval (CI) of small for gestational age (SGA) from polluting cooking fuel exposure after removal of single study result (leave-one-out analysis).

| Study omitted | Estimate | Lower CI | Upper CI | *I*^2^ |
| --- | --- | --- | --- | --- |
| Tielsch et al (2009) | 1.51 | 1.08 | 2.11 | 87.8 |
| Wylie et al (2014) | 1.56 | 1.19 | 2.05 | 89.0 |
| Yucra et al (2014) | 1.43 | 1.09 | 1.88 | 89.1 |
| Jiang et al (2015) | 1.50 | 1.13 | 1.99 | 89.4 |
| Jiang et al (2015) | 1.50 | 1.12 | 2.01 | 89.4 |
| Fleisch et al (2020) | 1.52 | 1.14 | 2.03 | 89.3 |
| Fleisch et al (2020) | 1.53 | 1.15 | 2.03 | 89.3 |
| Fleisch et al (2020) | 1.46 | 1.10 | 1.94 | 89.4 |
| Weber et al (2020) | 1.48 | 1.12 | 1.95 | 89.4 |
| Paudel et al (2020) | 1.48 | 1.10 | 1.99 | 89.4 |
| Hussein et al (2020) | 1.47 | 1.12 | 1.94 | 89.4 |
| Hussein et al (2020) | 1.47 | 1.12 | 1.94 | 89.4 |
| Chaudhary et al (2021) | 1.26 | 1.14 | 1.39 | 15.9 |
| Vakalopoulos et al (2021) | 1.46 | 1.10 | 1.93 | 89.3 |
| Pan et al (2022) | 1.50 | 1.11 | 2.02 | 89.4 |
| Pan et al (2022) | 1.51 | 1.12 | 2.04 | 89.3 |

Figure S1. Egger's funnel plot of low birth weight (LBW).

Figure S2. Egger's funnel plot of small for gestational age (SGA).

Figure S3. Egger's funnel plot of stillbirth.

Figure S4. Egger's funnel plot of preterm birth (PTB).
